# Developing a Personalized Integrative Obesity-Coaching Program: A Systems Health Perspective

**DOI:** 10.3390/ijerph19020882

**Published:** 2022-01-13

**Authors:** Sander M. Brink, Heleen M. Wortelboer, Cornelis H. Emmelot, Tommy L. S. Visscher, Herman A. van Wietmarschen

**Affiliations:** 1 Department of Preventive Rehabilitation, Vogellanden Center of Rehabilitation Medicine & Special Dentistry, 8001 BB Zwolle, The Netherlands; c.h.emmelot@vogellanden.nl; 2 Department Microbiology and Systems Biology, The Netherlands Organization for Applied Scientific Research (TNO), 3700 AJ Zeist, The Netherlands; heleen.wortelboer@tno.nl; 3 Staff Office Education and Research, Hanze University of Applied Sciences, 9747 AS Groningen, The Netherlands; t.l.s.visscher@hanze.nl; 4 Department of Nutrition and Health, Louis Bolk Institute, 3981 AJ Bunnik, The Netherlands; h.vanwietmarschen@louisbolk.nl

**Keywords:** obesity, systems thinking, personalized integrative coaching program, collaborative transdisciplinary approach

## Abstract

Current obesity management strategies are failing to achieve sustainable and favorable long-term results. We propose a more personalized, dynamic, and systemic perspective on the interactions of key determinants and coaching advice on obesity. The aim of this study was to use a systems view on overweight, complexity science, and a transdisciplinary process to develop a five-year personalized integrative obesity-coaching and research program. Managers, medical specialists, clinical psychologists, dieticians, physical- and psychomotor therapists, and lifestyle coaches aligned their perspectives and objectives with experts in systems thinking and systems biology. A systems health model of obesity was used to identify the causal relations of variables with the most influence on obesity. The model helped to align and design a personalized integrative obesity-coaching program and to identify the key variables to monitor the progress and to adjust the personalized program, depending on the goals and needs of the participant. It was decided to use subtyping of participants by a systems biologist, based on traditional Chinese medicine symptoms, as a novel method to personalize the intervention. The collaborative transdisciplinary approach based upon a systems view on obesity was successful in developing a personalized and adaptive five-year obesity-coaching and research program.

## 1. Introduction

Overweight and obesity have become the most important public health concerns due to their association with non-communicable diseases (i.e., type 2 diabetes mellitus, cardiovascular- and psychological disorders), COVID-19, and related economic consequences [1,2]. Unfortunately, the prevalence of overweight and obesity is still rapidly rising in developed and developing countries [1]. The worldwide prevalence of obesity nearly tripled between 1975 and 2016 [1]. The WHO estimated in 2016 that 39% of the adults worldwide were overweight and approximately 13% were obese. In The Netherlands, half of the adult citizens are currently overweight, and almost 14% of those were struggling with obesity in 2020 [3]. It is estimated that the percentage of adults in The Netherlands who are overweight will increase to 62% in 2040 [4]. Over the past decades, the prevalence of morbidity, impairments and disability as a result of obesity is increasing in clinical practice [5]. Apart from diabetes mellitus type II (which may result in cardio- and/or cerebrovascular disease, renal failure, blindness, and major amputation), the prevalence of overuse syndromes (i.e., osteoarthritis and tendinitis), lack of fitness, and dyspnea are increasing as well. Patients complain about feeling shame and reduced self-esteem. All these sequelae lead to an increasing demand for health insurance, sickness benefit, and employment compensation.

Lifestyle-related factors are the dominant causes of becoming overweight or obese, besides ageing of the population, genetic predisposition, and medical treatment [6,7]. Both overweight and obesity can be prevented and reduced by adopting a healthier lifestyle [8]. Lifestyle interventions are efficient and safe strategies to manage obesity. Combined lifestyle interventions are defined as a form of treatment that targets diet, physical activity, and at least one other lifestyle component [9,10]. However, poor attendance and high attrition rates of existing lifestyle interventions limit treatment effectiveness and health outcomes [9]. Reported results of current lifestyle intervention programs are often biased towards only those highly motivated people who are able to continue the program for two years. It is well known how difficult it is to support people adopting sustained healthy lifestyle habits for a longer period of time [9,11,12].

To date, current healthcare strategies have not resulted in a reduction in the number of people with overweight and obesity in society. This is presumably the result of a general conception that overweight and obesity are (mainly) the result of the imbalance between calorie intake and calories burned [13]. This conception also implies that overweight and obesity are the result of personal problems or failure. However, this completely disregards both many and large inter-individual differences in physical, psychological, social, behavioral as well as environmental and political factors that play a tremendous role in the development of an overweight and obese society [5,7,14]. Clearly, overweight and obesity cannot merely be reduced to simply advising people to eat less or exercise more; it is a complex issue involving many domains of science, behavior, society, economy, and policy [5,15,16,17]. Therefore, a different approach is needed to deal with overweight and obesity healthcare, namely an integrative program primarily focused on sustainable mental and lifestyle changes and developed from a complexity science perspective [18,19]. Such an approach requires a systems view on overweight, a view including multiple physical, mental, social, behavioural, genetic, and environmental factors interacting in a non-linear manner.

Complexity science in health (care) is the study of dynamic processes of a large number of biological, psychological, social and behavioral determinants, which fits very well with such a systems view on overweight. These determinants contribute to unhealthy behavior and physiology causing overweight and obesity. The key determinants may be different for each individual, and there are many interactions between these determinants with various strengths and speeds over time [19,20]. Overweight and obesity can be conceptualized as the outcome of a multidimensionally complex problem, with sudden changes in patterns of behavior, habits, social connections, events, physiology as well as slow deterioration of these patterns over time, which can result in relatively stable adaptation strategies [21,22]. Hence, (complex) interventions should focus on destabilizing unhealthy stable states, pushing these towards healthier stable states. For each individual, with a personal pattern of organization, there are particular types of interventions that might cause such a destabilizing effect. A systems view of the problem is therefore needed to figure out which particular intervention works for which person [23].

Interestingly, such a systems view on health has been developed and practiced for thousands of years in China [24]. Chinese medicine is especially focused on observing and treating patterns of symptoms, which include physical, mental, behavioral, and nutritional aspects. According to Chinese medicine, overweight and obesity are patterns of symptoms which are an expression of underlying imbalances in the person. Five of those patterns represent five distinct subtypes of people with overweight with very different biopsychosocial characteristics. Several Chinese diagnosis-based subtypes have been validated using analytical chemistry methods, revealing relationships between Chinese patterns of symptoms and biological mechanisms [17,25,26]. A description of the Chinese subtypes for obesity is shown in Table 1 based on two textbooks of Chinese Medicine [27,28]. In a Chinese medicine treatment approach, overweight itself is not treated as such, but the subtypes or patterns that are the cause of the imbalance in the person. For each of these patterns, specific advice on nutrition, manner of preparing food, exercises, and herbal medicines is available. Therefore, Chinese subtyping may offer new insights for a more personalized treatment of overweight and obesity.

In short, to develop a long-term effective and personalized integrative obesity-coaching program, we argue that a systems approach to obesity is needed. This paper presents a description of the development of a long-term personalized integrative obesity-coaching program with a highly adaptive character, targeting adults, which is based on a theory-driven group modelling approach involving the potential target group of patients and involved professionals. In follow-up studies, the adaptive program will be evaluated. We expect that a more personalized integrative coaching of obesity improves compliance and effectiveness, resulting in a sustainable long-term lifestyle change.

## 2. Materials and Methods

### 2.1. Theoretical Approach: Systems Health

The systems view on health was ‘translated’ into a multidisciplinary treatment plan for people with overweight [19]. In order to perform this translation, a causal loop diagram (CLD) was developed using the system dynamics methodology [29]. Constructing such a CLD allowed the integration of knowledge from a variety of scientific disciplines including medical biology, systems biology, food and nutrition, analytical chemistry, microbiology, psychophysiology, movement sciences, immunology, social sciences, and psychology [30,31]. 

The systems health model was a semi-quantitative CLD built with the TNO proprietary software MARVEL 3.2.11.4 [20,32]. Between 8 and 10 scientists with backgrounds in the above-mentioned scientific disciplines came together during three 4 h group model building sessions over a period of 1 year. During the group model building sessions, a moderator with a background in systems dynamics constructed the model with the MARVEL software on a screen for all participants to see. The first step in the model building process was the identification of the most important variables for health. Secondly, the relationships between those variables were identified, followed by an estimation of the strengths and speeds of these relationships. Key factors and their causal relations were confirmed with data captured from scientific literature. To achieve this, a non-systematic, iterative literature search was performed with a focus on original research reports and literature reviews on overweight and obesity that were published as of the year 1980 in international peer-reviewed journals. As we progressed into the systems health model development fine-tuning phases (see below), we repeatedly searched for additional literature whenever new gaps in the CLD were identified.

The model was then used to simulate various chronic stress- and overweight-related scenarios using the MARVEL software. Simulations revealed the variables with the most influence on health, which then supported the development of the lifestyle program. Then, a pragmatic set of measurements and instruments for all of those variables were identified by the experts.

### 2.2. Intervention Design and Participants 

The initiative to develop a multidisciplinary obesity coaching program started with the assumption that a center of rehabilitation medicine is a suitable organization for the development of a new comprehensive treatment of obesity. Medical specialists in physical medicine and rehabilitation (PM&R) (working at the rehabilitation center from a holistic concept) are experts in designing comprehensive, patient-centered treatment programs and are ultimately responsible for this program. They utilize time-tested treatments to maximize function and quality of life for their patients. Treatments aim to enhance and restore functional ability and quality of life for those with physical impairments or disabilities. A center of rehabilitation medicine provides an infrastructure for implementation and execution of treatment programs.

The CEO of Vogellanden, Center of Rehabilitation Medicine & Special Dentistry (Vogellanden) and a Physical Medicine and Rehabilitation physician (PM&R physician) of Vogellanden and the Isala Hospital (Zwolle, The Netherlands) increasingly realized that a healthy lifestyle is important to reduce the medical consequences of overweight and obesity. They also recognized the limitations of existing monodisciplinary strategies and comprehensive treatments in the management of obesity in the long run. Therefore, they contacted the Department of Systems Biology of The Netherlands Organisation for applied scientific research (TNO) in 2014, well known for its extensive knowledge of complex systems, systems thinking, and both Chinese and Western medicine [17,19,21,24,29]. Both institutes agreed to create a long-term multidisciplinary and personalized intervention program for adults with obesity in The Netherlands, taking into account the (changing) needs and capabilities of the participant. The aim of this new intervention was to achieve a sustainable healthier lifestyle, resulting in a better quality of life, reduction of overweight, and health benefits for people with obesity. 

Subsequently, based on the Dutch guidelines of obesity treatment [33,34] and the results of the system health model for overweight, (healthcare) professionals were recruited for the new comprehensive intervention. Meetings were organized to determine the key-elements of the intervention to discuss and using a mutual agreement procedure to determine the elements of the coaching and research program. There was skepticism against implementing Chinese medicine principles. After extensive discussion, consensus was found. We agreed that recognizing the Chinese subtypes with accompanying nutrition and exercise advice would be implemented only when they did not interfere with usual recommendations.

In- and exclusion criteria and recruitment of the potential participants, informed consent form, format of the intervention, and outcome measures with a time schedule were determined. Furthermore, the role of the professionals, treatment frequency, and treatment duration, individual and/or group sessions, and conditions (which the program must meet) were discussed. It was also discussed how the participants and their environment (i.e., family, workplace) could be actively involved in the program. Finally, the team agreed that the program would start in 2015 and that it would be further developed and adapted based on the experiences of healthcare professionals and participants, and new (scientific) insights.

## 3. Results

### 3.1. Systems Health Model of Obesity

**Figure 1:**
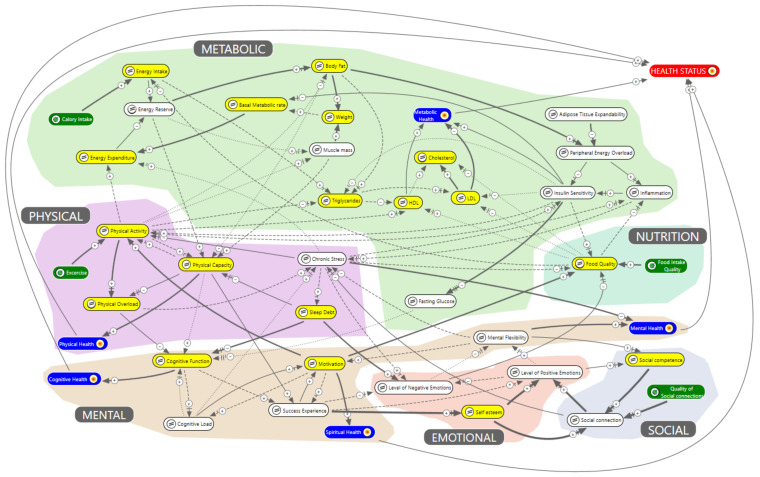
Systems health model of obesity. Variables are connected with arrows indicating the direction of the relationship. The thickness of the arrows indicates the strength of the relationship, small lines near the head of the arrow indicate the speed of the relationship, a plus or minus indicates a positive (reinforcing) or negative (counteracting) relationship. The model consists of several domains identified by colored clouds and by the grey labels: metabolic, physical, nutrition, mental, emotional, and social. The variables in yellow are the ones that will be monitored. Green variables can be changed by the individual; the blue and red ones are health goals [32].

The group modelling revealed six main domains of obesity, i.e., mental, emotional, physical, metabolic, nutrition, and social, which all determine the overall health status of an individual. These six domains are visualized in our systems health model of obesity, as presented in Figure 1.

### 3.2. Organizational Structure of the Personalized Integrative Obesity-Coaching Program

According to the experts and literature, chronic stress and motivation appeared to be key factors in the development of obesity and thus a reason to include a systems biologist and psychologist in the program. The model also showed that for each domain (physical function, nutrition, metabolic, social, emotional, and mental), a healthcare professional should join the program design.

The choice of the healthcare professionals participating in the program was based upon the system health model. The design team decided that the program should consist of a multidisciplinary team, working under supervision of a PM&R physician. The PM&R physician will supervise the inclusion, the medical aspects of the treatment, the monitoring of parameters, and external communication. After initial assessment of a potential participant, the PM&R physician and a clinical psychologist will decide whether the potential participant can participate in the program. It was decided that every participant would be assigned one of the lifestyle coaches as a case manager. Lifestyle coaches will be responsible for their own expertise (nutrition, physical activity, stress, sleep, and environment) but will collaborate intensively with each other.

**Figure 2:**
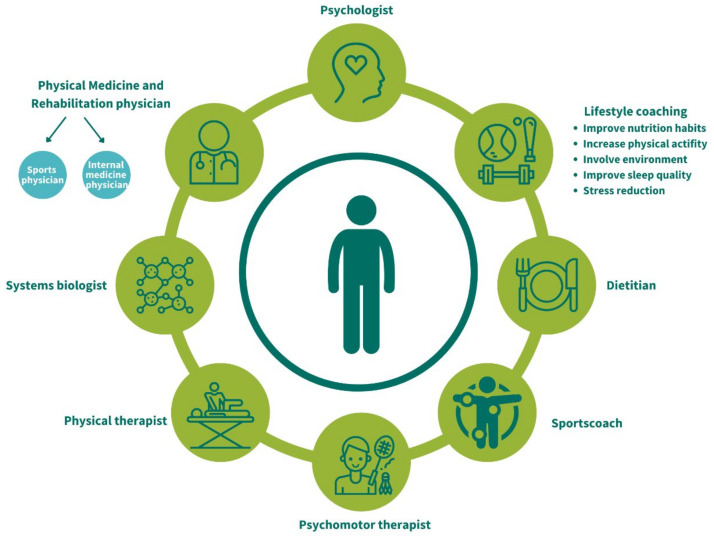
An overview of the healthcare professionals involved in the personalized integrative obesity-coaching program based on important factors from the system health model.

A systems biologist will be responsible for subtyping the participants in one out of five Chinese diagnostic symptom patterns, primarily intended to recognize these different subtypes, which will possibly help to further personalize the intervention. To be able to determine the subtype, participants will complete a questionnaire prior to consultation (see Supplementary Data). Optional expertise of a consultant in sports medicine and/or in internal medicine can be requested. Additionally, a physiotherapist and psychomotor therapist will be closely involved in the program. An overview of the involved healthcare professionals is shown in Figure 2. 

### 3.3. Conditions for Participating in the Program

The design team decided that the obesity-coaching program will be developed for people aged 18 years or above with obesity (BMI ≥ 30 kg/m^2^). Potential participants with pulmonary-, cardiovascular-, internal- and/or psychologic disorders that may interfere with the intervention will be excluded from the program. Prior to the start of the program, some participants will be selected for a maximum performance test in cases of: age > 40 years, proven pulmonary of cardiovascular risk factors, and/or serious doubt about physical capacities. Healthcare providers located in Zwolle and surroundings (i.e., general practitioners in the region and medical specialists for internal diseases working at the Isala Hospital) will be informed about the program. They will recruit potential participants and refer them to the Vogellanden center of rehabilitation medicine, special dentistry, and prevention (Zwolle, The Netherlands).

### 3.4. Ethics and Informed Consent

The Medical Ethics Committee of the Isala Hospital (Zwolle, The Netherlands) reviewed the evaluation protocol of the program (METC number: 15.0224). They concluded that further ethical approval within the scope of the Dutch Medical Research Involving Human Subjects Act (Central Committee on Research Involving Human Subjects (CCMO)) is not needed. All participants will sign an informed consent prior the start of the program.

### 3.5. The Contents of the Personalized Integrative Obesity-Coaching Program

The coaching program will be a highly adaptive long-term personalized lifestyle intervention for obese adults based upon the system health model for overweight. The aim of the program is to achieve, within a timeframe of five years, a sustainable healthier lifestyle of the participants by modifying behavior focusing on nutrition, physical activity, sleep and stress, while taking into account the changing needs and possibilities of the participant in his/her environment (i.e., family and work). Major goals are personal growth (the act of developing as an individual, in several areas including mental, physical, and emotional health) and meaning of life (which refers to people’s concerns with the core significance and purpose of their personal existence). Concrete additional goals of the program are improvements in quality-of-life scores, 5–10% sustainable weight loss, and subsequent improvement of general health. To achieve these goals individual coaching sessions, group meetings and social events will be organized. 

In a plenary session prior to the start of the program, the participant and a representative of our rehab center will both sign a covenant in which they commit themselves to the principles of the program and intended results. During the first twelve weeks after the start of the program, information based on individual goals, -needs, questionnaires and biomedical- and anthropometric characteristics will be put together and will be used to develop a personal (coaching) strategy for each participant. Therefore, the content of the intervention and coaching frequency may vary amongst participants. For example, completed food diaries will be used to provide dietary advice appropriate to the needs of the participants. The TCM subtype can also be taken into account when giving nutritional advice (e.g., Subtype I will be advised to eat a warm breakfast and high fiber foods).

In addition, participants will participate in at least 27 theme-sessions in the first three years of the program. During these group sessions, topics related to a healthy lifestyle will be discussed. These group sessions were divided into five blocks, and it was decided that the following main themes will be discussed: dietary behaviors and nutrition, sustainable behavior change, body experience and physical activity, sleep and stress, and mindful eating. In preparation for the meetings, participants will be asked to read the offered documents and complete homework assignments (i.e., writing a motivational letter to yourself in which questions such as “Why do you want to change?” and “What do you need to change?” are explored). Furthermore, workshops will be organized in which what is learned will be applied in practice (i.e., healthy cooking and interpreting food labels). Finally, a book club will be founded for those who are interested. During informal meetings, books about health and healthy lifestyle will be discussed. Participants can propose the title of a book themselves. Family and friends of the participants are regularly invited to these meetings and workshops in order to involve them in the program. 

Furthermore, participants will use a Vogellanden proprietary smartphone app (Vogellanden vitaal) in which recipes and informative videos will be shared. The app will also be used by participants to view their own progress by recording outcomes as measured by coaches (i.e., weight, waist circumference) and determined in a hospital laboratory (i.e., lipid profile), and personal goals. The app will also include an agenda (with upcoming sessions and meetings) as well as a forum for discussions and sharing of experiences. An important feature of the program is the buddy system, in which all participants will voluntarily be linked to another participant, a so-called ‘buddy’ [35]. Both ‘buddies’ are able to monitor and help each other, when they need support.

Changes towards a healthier lifestyle will be achieved by setting small goals and, when these goals are achieved, setting new small goals [36]. Progress will be frequently monitored by conducting interviews, allowing participants to complete questionnaires and by collecting biomedical and anthropometric data. Depending on the goals and the needs of the individual participants, they will participate in the coaching and research program for up to five years.

The procedure of running the program and the progress of each participant will be evaluated and discussed every 6–8 weeks with the entire team. In between these evaluations, coaches will be allowed to fine-tune their approach daily. As long as participants participate in the program, progress will be monitored at pre-arranged times. Depending on these evaluations and the measurements (including questionnaires), the treatment strategy will be determined. For example, for one participant, the emphasis will be on psychologist support, while for another participant, improving the diet will be the most important part of the program.

### 3.6. Monitoring Plan

The monitoring plan was also based on the results of the systems health model of obesity (Figure 1). The model shows the most important variables to measure during the lifestyle program in yellow. The health outcomes are the blue colored variables. Health status was chosen as a summary outcome measure for health, colored in red. The model consists of several domains identified by colored clouds and by the grey labels: metabolic, physical, nutrition, mental, emotional, and social.

The most important (yellow) variables for measuring the effects of the lifestyle program were: energy intake, energy expenditure, basal metabolic rate, body fat, weight, Hba1C, triglycerides, lipid profile, physical activity, physical overload, physical capacity, cognitive function, sleep debt, motivation, food quality, social competence, and self-esteem. Measurement instruments for each of those variables were assessed for possible application in practice during the lifestyle program. Table 2 shows the chosen selection of the measurement instruments per health domain. 

**Figure 3:**
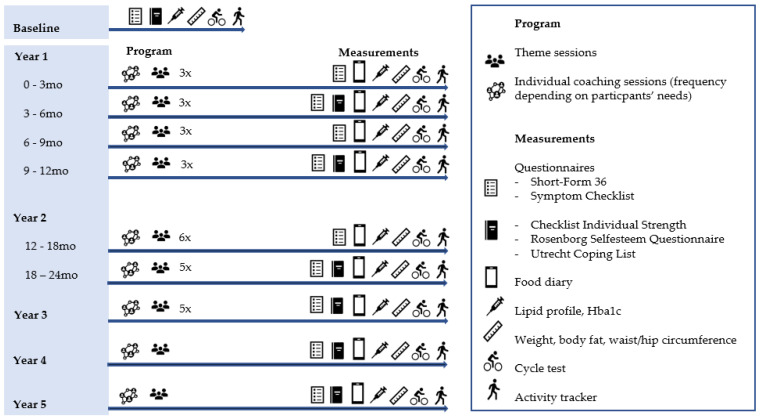
Description of the personalized integrative obesity-coaching program.

To gain insight into the changes in biomedical-, anthropometric-, and physical fitness, a standardized set of clinimetrics will be administered at pre-arranged times. A lipid profile (total cholesterol, HDL-/LDL-cholesterol and triglycerides) and HbA1c will be determined in the nearby hospital laboratory and registered in mmol/L. Weight (kg) and (visceral-) fat percentage will be measured with a four-point bioelectrical impedance device of Tanita RD-545 (Tanita Europe BV). Waist- and hip circumferences will be measured with a measuring tape in centimeters [37]. Maximal aerobic capacity (VO_2-max_) will be predicted based on the submaximal cycle ergometer aerobic fitness test according to the protocol of Åstrand (mL/kg/min) [38]. The average intake of calories, proteins, fat, and carbohydrates will be recorded with the Dutch smartphone food diary app ‘mijn eetmeter’. Participants will fill in this diary for five days a week (Wednesday to Sunday). Average physical (in)activity (minutes per day) will be registered with a Polar activity tracker (Ignite or A370) over 7 consecutive days. In addition, participants complete, using an online portal, a Dutch version of the Short-form 36 [43], Rosenberg self-esteem questionnaire [40], the Checklist Individual Strength [39], Utrecht Coping List [41], and Symptom checklist (SCL-90) [42]. To determine the subtype of the participant, prior the start of the program, a Chinese subtyping questionnaire is administered [27,28]. As long as participants participate in the program, the outcomes will be measured several times. The project team discussed the systems health model and, based upon their experiences, decided that data will be collected before the start and at three, six, nine, twelve, eighteen, twenty-four, thirty-six, forty-eight, and sixty months after the start of the program. Individual changes are expected in the first phase after the start of the program. For this reason, the project team decided that the measurements will initially take place every three months. The measurements after two years and beyond are mainly intended to assess whether participants do not relapse. An overview of the longitudinal coaching and research design is presented in Figure 3.

The first group of ten individuals will participate in an adaptive pilot study. The feasibility of this pilot study and the experiences of healthcare professionals and participants will be studied. The results of this pilot study, which will be one year ahead of the program, will be used develop the personalized integrative obesity-coaching program.

### 3.7. Evaluation and Analysis

In order to determine the effectiveness of the personalized integrative coaching program, the collected data will be analyzed at the level of the individual participants. First, we will investigate the effect of the different measures with a linear mixed-effects model. This model can be used per outcome measure to represent the differences between the time points per participant in a one-dimensional way. The effect of the various interventions is also taken into account. To show the effect on different scales, we will look at a visual representation on some scales on a spider-plot and on a three-dimensional axis. This will be compiled with a combination of analysis methods such as factor analysis/main component analysis, as well as the created content models. This makes it possible to visualize the displacement and to quantify the change on these axes. The axes are composed of categories identified by experts as important and validated scales on questionnaires. It is also important to map out the mutual relationships. Using the systems health model of obesity with the relationships according to the experts, we can use the questionnaires and measurements to convey what the relationships are within the data. We will use the questionnaires and measurements as a starting point to draw up two network models: one per coaching moment, and one that shows the effects over time. This will provide insights into the mutual relationships between the various risk factors and outcome measures.

## 4. Discussion

This paper presents a description of the development of a long-term personalized integrative obesity-coaching and research program with a highly adaptive character, targeting adults with obesity based on a systems dynamic model of health. The growing interest in interventions that take into account the multifactorial drivers of overweight and obesity was recently reported in a systematic review [44]. In recent years, various (combined) lifestyle interventions have been developed in The Netherlands and have been scientifically proven to be effective up to 2 years after the start of the intervention in people with overweight, obesity, and/or type 2 diabetes [45,46,47,48]. Many of these programs offer a multidisciplinary approach, according to a fixed protocol between 6 to 24 months. Besides nutritional advice and increasing physical activity, the interventions pay attention to improving health skills and sleep quality and reducing stress. In one such program, the Reverse type 2 diabetes program, the effectiveness was recently improved by closely involving partners and family members of the participants, similar to our obesity-coaching program. As discussed before, poor attendance and high attrition rates that limit treatment effectiveness and health outcomes of lifestyle interventions are reported [9]. Often only the limited results of only those who completed the intervention are presented, while it is known that it remains difficult to adopt healthier lifestyle habits for a longer period of time [12].

### Strengths and Limitations 

Due to the multifactorial drivers of obesity, a strength of the study is the integration of systems thinking and a transdisciplinary approach via the use of a systems health model of obesity to identify the causal relations of variables with the most influence on obesity. This helped to select and align all professionals towards collaboratively developing the adaptive obesity-coaching program. The model was used to identify the key variables to monitor the progress of the participants, which then supports further fine-tuning of the program. The second strength is that this program, in addition to the usual goals of lifestyle interventions (sustainable lifestyle change, weight loss, improvement of general health and quality of life), focuses on personal transformation and personal growth towards improvements to become a ‘better version of the individual’.

For each participant, the meaning of life plays an important role in this program. The third strength is the active (participatory) role of participants, their family, and their social environment, which will all be involved in the program.

Besides strengths, some limitations can also be identified. The design of the program is highly personalized and adaptive. Each individual is unique and will follow its own path. Therefore, the heterogeneous nature of the resulting data will not allow a standard experimental controlled design. However, whilst the outcomes of the described program can be compared with outcomes of other lifestyle interventions, direct comparison with a group control group (i.e., after usual care) will not be possible.

Actually, a longitudinal N = 1 study design will be followed. In addition, the personalized integrative approach makes the study very laborious, and in the first two years, a group of only 10 participants per year will be included. Information on the healthcare professionals, participants, and measurements will be used to develop the program continuously, taking into account the efficiency (e.g., limiting the number of sessions, using Vogellanden Vitaal app) and costs (e.g., discussing the program with Dutch healthcare insurance companies). Only when the program is fully evolved can we foresee that after 3 years of experience two or even more groups of participants can be included each year. Therefore, the recruitment of sufficient participants for a substantial evaluation of the effectiveness of the program will take some time.

Despite these limitations, the personalized integrative obesity-coaching program described in this paper has a unique and added value in its personalized approach and the 5 year period in which participants will be coached and monitored. In addition, due to the adaptive nature of the coaching program, participants will receive coaching when there is a need, instead of at fixed points in time. Finally, our coaching program will act as a learning community and will be continuously improved based on the experiences of participants, professionals, and the newest scientific knowledge.

## 5. Conclusions

This collaborative transdisciplinary approach based upon a systems view on obesity was successful and led to a new intervention for individuals with obesity. The result was a personalized and adaptive five-year obesity-coaching and research program. The project has been in progress since 2015. Eleven groups with approximately ten participants each have started the program so far. Dissemination of the first results is planned for early 2022.

## Figures and Tables

**Table 1:** Chinese subtypes of obesity divided into physical, mental, and food/metabolism characteristics based on two textbooks of Chinese medicine [27,28].

	Characteristics		
Subtype	Physical	Mental	Nutritional/Metabolism
I Spleen Deficiency with Damp	Weak fat tissue in front of the abdominal muscles; low muscle mass, having fatigue/drowsiness; unpleasant feeling in the chest and/or stomach area; sweating easily.	Gets worried quickly; foresees a number of problems.	Likes sweet food; limited appetite; does not feel thirsty; difficult digestion.
II Stomach Yin Deficiency	The same as subtype 1 combined with reflux.	The same as subtype 1 combined with an increased arousal.	Having a constant need to eat; difficult digestion.
III Damp Heat and Qi Stagnation	Solid muscle mass; excessive weight, particularly in the abdominal area; chest breathing; often having musculoskeletal disorders.	Dreamy; irritated easily; struggles with setting personal boundaries.	Thirsty; overeating; constipation.
IV Yin Deficiency	Accumulation of fat in the abdominal area and buttocks; tired, edema in lower extremity; aversion to cold; sometimes thyroid disorders.	Anxious; difficulty with recognizing personal boundaries; reduced body awareness.	Often eating normal amounts; sometimes poor appetite; irregular defecation.
V Liver Qi Stagnation	Looking bloated; accumulation of fat in the abdominal area; has little energy; increased stress hormones; vertigo.	Having a tendency to rationalize; think and worry; likes to be in control; urge to prove.	Eating quickly; dry mouth; reduced appetite; cramping feeling in abdomen; reflux; nausea.

**Table 2:** Selection of the measurements per health domain.

Health Domain	Measurements
Metabolic	Lipid profile, HbA1c, weight, body (visceral-) fat%, waist/hip circumference [37]
Physical	Maximal aerobic capacity [38], activity tracker
Mental	Checklist Individual Strength (CIS) [39]
Emotional	Rosenberg self-esteem scale (RSE) [40]
Social	Utrecht Coping List (UCL) [41]
Broad spectrum health measures	Symptom checklist (SCL-90) [42], Short Form 36 (SF-36) [43]
Nutrition	Food diary app ‘mijneetmeter.nl’

## Supplementary Materials

The following are available online at https://www.mdpi.com/article/10.3390/ijerph19020882/s1.
