# Supplementary material for: Developing a Personalized Integrative Obesity-Coaching Program: A Systems Health Perspective
Source: Int J Environ Res Public Health. 2022 Jan 13;19(2):882. doi: 10.3390/ijerph19020882 (PMC8775850; doi:10.3390/ijerph19020882)
Supplement: Supplementary file 1 [file ijerph-19-00882-s001.zip › ijerph-1460703-supplementary.pdf]

## System Biologist Questionnaire

This questionnaire will be used to identify sets of symptoms related to aspects of obesity. This information can be used to develop more personalized treatment strategies.

The questions will take approximately 15 minutes to complete.
Please complete all questions by circling the correct answer.
If no answer is possible, please indicate this. All information will be treated confidentially.

Best of luck to you!

| **Tiredness** | |
|---|---|
| I generally feel tired | yes – no |
| *How strong is this tiredness* | not strong – a little – moderate – strong – very strong |
| I am easily tired | yes – no |

| **Sleep** | |
|---|---|
| I have trouble falling asleep | yes – no |
| I often wake up at night | yes – no |
| I feel sleepy during the day | yes – no |
| I put my feet under the covers at night to cool off | yes – no |

| **Movement** | |
|---|---|
| After physical exertion I feel | very tired – tired – normal – better |
| I perspire easily with a little exertion | yes – no |

| **Digestion/Appetite** | |
|---|---|
| My appetite is | very poor – poor – normal – good – very good |
| I regularly have pain in the stomach | yes – no |
| I regularly suffer from abdominal bloating | yes – no |
| I regularly suffer from belching, burping and/or heartburn: | yes – no |
| | |
| Eating soothes my stomach when it feels restless | yes – no |
| I regularly have to gag | yes – no |
| | |
| My need to drink is | great – normal – small |
| I often have a dry mouth | yes – no |
| I often have a bitter taste in my mouth | yes – no |
| I often have bad breath | yes – no |
| I suffer from excessive saliva | yes – no |
| | |
| I need to urinate often | yes – no |
| What colour is your urine in general? | dark yellow – yellow – clear water |
| How is your stool in general? | diarrhoea – mushy – solid – hard |
| Does your stool alternate between soft and runny, and then again with constipation? | yes – no |
| After eating, I quickly feel hungry again | yes – no |

Supplementary data
Brink et al. Developing a personalized integrative obesity-coaching program: a systems health perspective.

| **Physical** | |
|---|---|
| **Do you regularly suffer from... ?** | |
| | |
| Headache | yes – no |
| *Applying pressure to the head:* | relieves the headache – aggravates the headache |
| Tinnitus | yes – no |
| Sneezing | yes – no |
| Burning/dry eyes or difficulty in focusing | yes – no |
| Sore/weak knees | yes – no |
| Low back pain | yes – no |
| Night sweats | yes – no |
| | |
| Shortness of breath | yes – no |
| Feeling of pressure on the sternum | yes – no |
| Feeling of having a lump in the throat | yes – no |
| An aversion to cold | yes – no |
| | |
| A languid/heavy feeling in the arms and legs | yes – no |
| Tiredness in the arms and legs | yes – no |
| Moisture in the lower legs | yes – no |
| Cold arms and/or legs | yes – no |
| Little strength in the arms and legs | yes – no |
| Cold back/buttocks/legs | yes – no |
| Feeling of having a hot head at the end of the afternoon | yes – no |
| | |
| **Pain** | |
| I am in pain | yes – no |
| *The pain can be described as* | stabbing, sharp and easy to locate – "nagging", diffuse and radiating |

| **Mental** | |
|---|---|
| I talk | little – normal – a lot |
| I have little motivation to do things | yes – no |
| I am dreamy during the day | yes – no |
| I am easily irritated | never – rarely – rather – very – very much |
| I often feel listless | yes – no |
| I worry and grind a lot | yes – no |

Supplementary data
Brink et al. Developing a personalized integrative obesity-coaching program: a systems health perspective.

| Questions for women | |
|---|---|
| My period is | too early - regular - too late |
| My period is painful | yes – no |
| I have mood swings around my period | yes – no |
| My period sometimes skips a month | yes – no |
| I have clear vaginal discharge | yes – no |
| I have less desire for sex | yes – no |

| Questions for men | |
|---|---|
| I have difficulty getting or keeping an erection | yes – no |
| I have less desire for sex | yes – no |
